# Stressful life events and relapse of psychosis: analysis of causal association in a 2-year prospective observational cohort of individuals with first-episode psychosis in the UK

**DOI:** 10.1016/S2215-0366(23)00110-4

**Published:** 2023-06

**Authors:** Sagnik Bhattacharyya, Tabea Schoeler, Marta Di Forti, Robin Murray, Alexis E Cullen, Marco Colizzi

**Affiliations:** a Department of Psychosis Studies, Institute of Psychiatry, Psychology and Neuroscience, King's College London, London, UK; b Department of Computational Biology, University of Lausanne, Lausanne, Switzerland; c Division of Insurance Medicine, Department of Clinical Neuroscience, Karolinska Institutet, Stockholm, Sweden; d Unit of Psychiatry, Department of Medicine (DAME), University of Udine, Udine, Italy

## Abstract

**Background:**

Despite accumulating evidence of an association between stressful life events and psychosis relapse, the extent to which this is a causal relationship remains unclear. We aimed to examine the association between exposure to, and number of, stressful life events after initial psychosis onset and psychosis relapse.

**Methods:**

In this 2-year prospective observational study, we recruited individuals with first-episode psychosis, aged 18–65 years, who presented to psychiatric services in south London, UK. Participants were assessed via interview, with additional data obtained from electronic clinical records. Stressful life events were recorded at psychosis onset and during the 2-year follow-up using a brief questionnaire that assesses 12 major life events. Psychosis relapse was defined as inpatient admission because of symptom exacerbation within 2 years from psychosis onset. We examined the time to first psychosis relapse and the number and length of relapses using survival and binomial regression analyses. We used fixed-effects regression and cross-lagged path analysis to examine the directionality of effects and control for unmeasured confounders.

**Findings:**

Between April 12, 2002, and July 26, 2013, 256 individuals with first-episode psychosis (100 [39%] female and 156 [61%] male; 16 [6%] Asian, 140 [55%] Black African or Caribbean, 86 [34%] White, and 14 [6%] mixed ethnicity) were recruited, with a mean age of onset of psychosis of 28·06 years (SD 8·03; range 17·21–56·03). 93 (36%) participants experienced at least one relapse during the 2-year follow-up. 253 individuals had all relevant data and were included in analyses. For people exposed to stressful life events after the onset of psychosis, the adjusted hazard (hazard ratio [HR] 2·60, 95% CI 1·63–4·16, p<0·0001), incidence (incidence rate ratio [IRR] 1·87, 1·24–2·80, p=0·0026), and length (IRR 2·53, 1·40–4·67, p=0·0011) of relapse were greater than for those who were unexposed. These relationships were dose dependent (HR 1·36; 1·09–1·69, p=0·0054; incidence IRR 1·26, 1·02–1·53, p=0·023; length IRR 1·52, 1·12–2·12, p=0·0028). Adjusted fixed-effects models showed a higher (odds ratio [OR] 3·82, 1·82–8·00, p=0·0004) and dose-dependent (OR 1·62, 1·18–2·21, p=0·0028) risk of relapse when stressful life events preceded relapse compared with the period when they did not. Cross-lagged path analysis confirmed an effect of stressful life events on the number of subsequent relapses (β=0·66, p=0·0055) that was dose dependent (β=0·29, p=0·029), but it did not show an effect of relapses on subsequent risk or number of stressful life events.

**Interpretation:**

These results provide converging evidence of a causal effect of stressful life events on the risk of relapse in psychosis. They suggest that there is a need to develop interventions at the individual and health-service level that could mitigate the harmful effects of stressful life events.

**Funding:**

National Institute for Health Research, UK.

## Introduction

A substantial proportion of individuals diagnosed with a first episode of psychosis will present with another episode in their lifetime,^1^ with poor clinical^2^ and psychosocial outcomes,^3^, ^4^ and increased health-care costs in those who relapse compared with those who do not.^5^, ^6^ Therefore, it is important to identify risk factors for relapse that individuals could recognise and monitor themselves and that might help to develop targeted interventions. Consistent with evidence that individuals who have been exposed to stressful life events have an increased risk of developing psychosis,^7^ stressful life events might also increase the risk of relapse in those with established psychosis.^8^, ^9^ However, the existing evidence is not easy to interpret.^10^ Although stressful life events occurring after the onset of psychosis have been linked to relapse of psychosis,^10^ methodological caveats limit the comparability of results across studies, hindering any attempt to draw firm conclusions.^10^ In particular, the confounding effects of illness stage and heterogeneity, and relevant sociodemographic and clinical variables (eg, medication adherence, substance use, and illness severity at onset) were not taken into account in most studies so far.^10^ Although we have addressed some of these limitations in a 2023 analysis,^11^ it remains to be determined whether the association between stressful life events and subsequent relapse of psychosis could be merely a reflection of shared genetic and environmental factors increasing the risk of both; systematic differences between people with psychosis who did and did not experience stressful life events could underlie the association observed.

**Research in context**

**Evidence before this study**

In October, 2020, we published a systematic review in which we reappraised the effect of being exposed to stressful life events on the risk of relapse in patients with psychosis. Relevant studies were identified by systematically searching PsycINFO, MEDLINE, and Embase from inception to Jan 8, 2020, using a combination of search terms for describing the exposure, the outcome of interest (relapse of psychosis), and the study population (people with psychosis). Data were gathered from 23 studies (2046 participants) comparing relapse outcomes in the following groups: people who had psychosis, people who were in remission, and healthy controls. 18 studies found a significant positive association between stressful life events and psychotic relapse, and five studies found a non-significant association. Methodological caveats were identified across the reviewed studies, including not considering the confounding effect of illness stage and heterogeneity as well as relevant sociodemographic and clinical variables (eg, medication adherence, substance use, and illness severity at onset). Furthermore, the methods used in previous studies to evaluate the association between stressful life events and relapse of psychosis did not allow for a causal interpretation.

**Added value of this study**

To the best of our knowledge, this is the first attempt to apply stringent causal inference methods to the key outstanding question regarding this association: whether stressful life events occurring after the onset of psychosis are causally associated with risk of subsequent relapse. We combined different approaches to assess temporality (prospective design and counting stressful life events that pre-dated outcome), directionality, the dose–response effect (whether the magnitude of effect varies with the number of stressful life events), and the confounding effect of unmeasured factors that do not change over time, such as shared genetic or environmental factors, in a large sample of people with first-episode psychosis. Participants were homogenous in terms of illness stage and were followed up for 2 years, an important period for potential predictors of relapse. Results showed a causal and dose-dependent effect of stressful life events on psychotic relapse, after controlling for a range of important confounders (eg, medication adherence, substance use, and disorder severity). Our findings were consistent across different outcome measures (eg, risk and number of relapses, length of relapse, and time until relapse). We excluded the possibility of reverse causation (psychosis relapse increasing the risk of subsequent stressful life events) or shared susceptibility between psychosis and stressful life events underlying their association.

**Implications of all the available evidence**

Exposure to stressful life events after the onset of psychosis is associated with a significantly poorer outcome of the disorder, which gets worse as a function of the number of stressful life events to which individuals with first-episode psychosis are exposed. This dose-dependent association is unlikely to be a result of genetic and environmental confounding or psychosis relapse increasing the occurrence of stressful life events. Thus, interventions at the individual health-service level for those who have experienced psychosis should aim to reduce the detrimental effects of stressful life events as and when they occur. Interventions could also aim to educate individuals with psychosis and their carers or families, after the onset of psychosis, to help mitigate the harmful effects of subsequent stressful life events.

The possibility of reverse causation in the context of stressful life events and psychosis relapse further complicates the interpretation of existing results. For example, relapse might increase the risk of separation from a partner or loss of employment. Ten of the 18 studies identified in our 2020 review,^10^ which reported an association between stressful life events and relapse of psychosis, indicated a temporal association, with stressful life events increasing the risk of subsequent relapse. To the best of our knowledge, none of these studies specifically tested the possibility of reverse causation (ie, whether relapse of psychosis increases the risk of subsequent exposure to stressful life events).

Here, we attempt to address these questions systematically by combining multiple inferential approaches. To specifically address the issue of temporality, we used a prospective design to investigate the effects of stressful life events (occurring after the onset of psychosis) on the risk of subsequent relapse within 2 years after onset of psychosis, accounting for measured potential confounding factors. We included sociodemographic and clinical factors (eg, alcohol and other drug use, age of onset, and illness severity at onset), and medication adherence, one of the strongest risk factors for relapse after a first episode of psychosis.^12^ Because up to one in two individuals with psychotic disorders will present with a relapse severe enough to require hospital admission within the first 2 years of their first-episode psychosis,^1^ this is a particularly important period for potential predictors of relapse. We examined whether the effects of stressful life events were consistent across a range of outcome measures and we tested for a dose–response relationship, an important criterion linked to causal association^13^ across these measures. We also applied fixed-effects analysis of longitudinal data,^14^, ^15^, ^16^ a causal inference approach that allows one to control for the effects of unmeasured confounders that do not change over time, such as shared genetic and environmental factors, as well as observed confounding factors that do change over time. Finally, we examined the question of directionality of association—whether stressful life events predict subsequent relapse of psychosis or vice versa (ie, reverse causation)—with use of a cross-lagged path analysis approach. We predicted that stressful life events occurring after the onset of psychosis would be associated with a significant risk of subsequent admission to hospital in a dose-dependent manner and that stressful life events would predict the risk of subsequent admission to hospital but not vice versa.

## Methods

### Study design and participants

In this prospective cohort study, we recruited individuals with first-episode psychosis presenting to psychiatric services in the catchment area of south London, UK (London boroughs of Southwark, Lambeth, and Croydon). Eligible individuals were experiencing a first episode of non-organic psychosis (non-affective [ICD10 codes F20–F29]) or affective [F30–F33] psychosis) and were aged 18–65 years, without any exclusion for comorbid conditions. Participants were followed up for 2 years. After providing written informed consent, individuals with first-episode psychosis were assessed twice, at the onset of psychosis via a face-to-face interview, and then at follow-up via face-to-face or telephone interview. At both assessment phases, additional data were obtained via a review of clinical records stored in the electronic patient journey system of the South London & Maudsley National Health Service (NHS) Foundation Trust. Study ethical approval was obtained from the South London and Maudsley NHS Foundation Trust and Institute of Psychiatry Local Research Ethics Committee (05/Q0706/158).

### Procedures

Stressful life events were assessed via interview using a brief life events questionnaire (the List of Threatening Experiences questionnaire)^17^ at onset and follow-up by trained study researchers (undergraduate or postgraduate students in psychology, mental health studies, or psychiatric research, and psychiatrists). This measure assesses the exposure to and emotional and stressful impact of 12 severe or major life events, and records the date on which they occurred. It has been used in studies of people with psychosis^18^, ^19^ and shown to exhibit high validity and reliability.^20^, ^21^ Post-onset stressful life events were quantified based on the follow-up assessment. For this study, the time frame for stressful life events was the 2-year follow-up period. The temporal order of life events and relapse events was ascertained based on the recorded date of occurrence of the stressful life event and the start date of the relapse event (hospital admission).

Psychosis relapse was defined as inpatient admission because of symptom exacerbation within 2 years from psychosis onset,^16^ a widely accepted^22^ and reliable relapse measure.^23^ Admission characteristics, including number, duration, and legal status (voluntary or involuntary), were extracted from electronic clinical records using the WHO Life Chart Schedule.^24^ In those who relapsed, only stressful life events that preceded relapse were counted, while in those who did not relapse, all stressful life events occurring during the 2-year study period were counted. Potential confounders assessed included sex (female or male), age of onset, relationship status (in a relationship or not in a relationship), ethnicity (White, Asian, Black African or Caribbean, or mixed ethnicity), other drug use (non-use, experimental, or regular), alcohol use (no use or user), cannabis use (former, never, intermittent, continued hash-type, continued regular skunk-type, or continued heavy skunk-type), nicotine use (no use, intermittent, or continued), care intensity at onset (community mental health team, crisis team, non-compulsory admission, or compulsory admission), medication adherence (regular compliance, irregular compliance, or poor compliance), and onset diagnosis (non-affective psychosis or affective psychosis). Further details are in the appendix (pp 2–5).

### Outcomes

The primary endpoint of interest was relapse of psychosis during the 2-year follow up. We examined: risk of relapse; number of relapses (cumulative number of hospital admissions after the onset of psychosis); length of relapse (cumulative number of months spent in hospital after onset of psychosis, not including time spent in hospital as part of the first episode); and time to first relapse (consecutive number of months without experiencing a relapse, with those not relapsing after onset allocated a time of 24 months).

### Statistical analysis

Data analysis was performed using R version 4.0.3 (survival, survminer, MASS, lme4, and lavaan packages; appendix pp 5–8). Separate survival analyses were carried out to investigate the effect of any stressful life events (yes or no), and the total number of such stressful life events occurring, between psychosis onset and relapse on the time to first relapse using Cox proportional hazards regression in a multivariable model controlling for the confounders.^16^ Because the proportional hazards assumption was violated at different levels of cannabis use, the model was stratified by cannabis use. A Kaplan-Meier plot was used to depict unadjusted survival data. Multiple negative binominal regression analyses were done for number and duration (assessed in months) of relapses. Medication adherence was included in separate regression models because this information was available only for the subset who were prescribed antipsychotics.

To investigate whether the effect of stressful life events on psychosis relapse was explained by unmeasured confounders varying across individuals but not over time (eg, familial or genetic factors, duration of untreated psychosis, or premorbid adjustment), we used fixed-effect logistic regression analyses for risk of relapse (yes or no) and fixed-effect negative binomial regression analyses for the number of relapses. We estimated the effect of exposure to, and number of, stressful life events on the likelihood of or number of relapses during the period when the individual was exposed to stressful life events compared with when the same individual was not exposed to stressful life events. We examined this in both simple (unadjusted analysis) and multiple regression models (adjusted for cannabis use, other drug use, and medication adherence). Unlike between-person estimates from conventional regression analyses, fixed-effects regression estimates are within-person, which can account for potentially confounding unmeasured personal characteristics.

To investigate reverse causation, we estimated competing fixed-effect regression models, testing for the effect of relapse on stressful life events. Furthermore, we estimated cross-lagged autoregressive path models to examine the directionality of association between relapse and stressful life events. The number of relapse events (number of relapses in year 1; number of relapses in year 2) was treated as the dependent variable and stressful life event exposure (yes or no in year 1; yes or no in year 2) or number of stressful life events (number of events in year 1; number of events in year 2) were treated as the independent variables (in separate models) to examine direct causation (stressful life events predicting subsequent relapse) and vice versa to examine reverse causation (relapse predicting subsequent stressful life events). Models controlled for medication adherence, cannabis use, other drug use, and pre-onset stressful life events. Model goodness of fit was assessed using root mean square error of approximation (values ≤0·05 indicating good fit) and comparative fit index (values >0·95 indicating good fit) and models with good fit indices are reported. As a first step, a saturated path model including all paths to endogenous variables was specified, followed by a more parsimonious path model including only statistically significant (p≤0·05) paths.

### Role of the funding source

The funder had no role in study design, data collection, data analysis, data interpretation, or writing of the report.

## Results

Between April 12, 2002, and July 26, 2013, a total of 256 individuals with first-episode psychosis (100 [39%] female and 156 [61%] male; 16 [6%] Asian, 140 [55%] Black African or Caribbean, 86 [34%] White, and 14 [6%] mixed ethnicity) were recruited, with a mean age of onset of psychosis of 28·06 years (SD 8·03; range 17·21–56·03). Follow-up assessments were completed up to Sept 23, 2015 (appendix p 14). No statistical differences in terms of sociodemographic and clinical characteristics at baseline were detected between participants and those who declined to be followed up and were therefore not included in the study (n=133), even when clustered by relapse status, and no differences were detected in their risk of relapse.^16^ Data on relationship status at onset were not available for three of 256 participants and they were excluded from analyses, leaving 253 participants for all analyses (except n=240 for analyses including medication adherence; data on medication adherence were not available for the remaining participants). Follow-up data up to 2 years from onset were available for all participants included in the analyses (n=253). Study sample baseline characteristics have been reported previously^16^ and are summarised in the appendix (p 15). 197 (78%) of 253 participants were admitted to hospital within 1 month of the onset of psychosis, of whom 117 (59%) were admitted as a compulsory admission. Within 2 years of psychosis onset, 92 (36%) of 253 individuals experienced at least one relapse of psychosis requiring hospital admission. The highest number of relapses recorded in the study period was three, with the longest hospital stay lasting 14·8 months. We found no effect of exposure to stressful life events (or number of stressful life events) before the onset of psychosis on the risk of subsequent psychosis relapse over the first 2 years after psychosis onset (appendix p 16).

**Figure 1:**
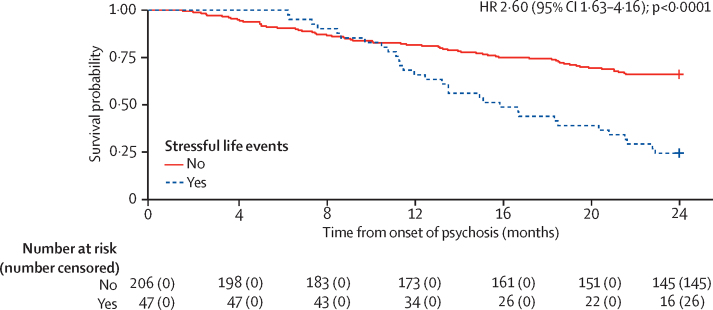
Kaplan-Meier estimates of the probability of psychosis relapse in individuals with and without post-onset stressful life events

Individuals who had experienced at least one stressful life event after psychosis onset (47 [19%] of 253) had a significantly higher hazard of experiencing a subsequent relapse of psychosis over the first 2 years after psychosis onset compared with those who did not experience any stressful life events (hazard ratio 2·60; 95% CI 1·63–4·16; p<0·0001), after controlling for several sociodemographic factors (sex, ethnicity, and relationship status) and clinical factors (psychosis diagnosis, intensity of care at onset, and alcohol, nicotine, and other drug use; with analysis stratified by cannabis use; table 1; unadjusted Kaplan-Meier survival curves are shown in figure 1). Including medication adherence in the model, while still controlling for sociodemographic and clinical factors, did not substantially change the results (2·60; 1·56–4·34; p=0·0003). Furthermore, a significant association was found between the number of stressful life events experienced and the hazard of subsequent relapse (1·36; 1·09–1·69; p=0·0054) after controlling for sociodemographic and clinical factors; after adjusting for medication adherence, this association had a similar magnitude (1·33; 1·06–1·68; p=0·015).

**Table 1:** Effect of stressful life events after onset of psychosis on hazard of subsequent relapse as indexed by hospital admission

		**Any stressful life events: hazard of relapse (n=253)**		**Any stressful life events: hazard of relapse (with medication adherence; n=240)**		**Number of stressful life events: hazard of relapse (n=253)**		**Number of stressful life events: hazard of relapse (with medication adherence; n=240)**	
		HR (95% CI)	p value	HR (95% CI)	p value	HR (95% CI)	p value	HR (95% CI)	p value
Any stressful life events before first relapse									
	No	1 (ref)	..	1 (ref)	..	..	..	..	..
	Yes	2·60 (1·63–4·16)	<0·0001	2·60 (1·56–4·34)	0·0003	..	..	..	..
Number of stressful life events before first relapse		..	..	..	..	1·36 (1·09–1·69)	0·0054	1·33 (1·06–1·68)	0·015
Age of onset, years*									
	<22	1 (ref)	..	1 (ref)	..	1 (ref)	..	1 (ref)	..
	22 to <25	0·98 (0·72–1·35)	0·91	1·0 (0·71–1·39)	0·97	0·99 (0·72–1·36)	0·96	1·02 (0·73–1·42)	0·92
	25 to <30	0·64 (0·01–67·1)	0·85	0·48 (0·00–64·9)	0·77	0·66 (0·01–67·9)	0·86	0·41 (0·00–53·4)	0·72
	30 to <39	4·96 (0·00–865 241)	0·79	9·43 (0·00–3 036 885)	0·73	4·25 (0·00–677 502)	0·81	12·6 (0·00–3 541 877)	0·69
	≥39	0·19 (0·00–2961)	0·74	0·13 (0·00–3175)	0·70	0·23 (0·00–3129)	0·76	0·12 (0·00–2402)	0·67
Sex									
	Female	1 (ref)	..	1 (ref)	..	1 (ref)	..	1 (ref)	..
	Male	0·75 (0·47–1·21)	0·24	0·59 (0·36–0·98)	0·043	0·72 (0·45–1·16)	0·18	0·56 (0·34–0·92)	0·023
Ethnicity									
	White	1 (ref)	..	1 (ref)	..	1 (ref)	..	1 (ref)	..
	Asian	1·99 (0·73–5·44)	0·18	1·47 (0·50–4·36)	0·48	1·98 (0·73–5·39)	0·18	1·41 (0·48–4·19)	0·53
	Black African or Caribbean	2·30 (1·27–4·15)	0·006	2·11 (1·15–3·90)	0·016	2·23 (1·24–4·01)	0·0076	2·04 (1·11–3·74)	0·022
	Mixed	2·09 (0·77–5·72)	0·15	2·17 (0·79–6·00)	0·13	1·99 (0·74–5·39)	0·17	1·91 (0·70–5·25)	0·21
Other drug use									
	Non-use	1 (ref)	..	1 (ref)	..	1 (ref)	..	1 (ref)	..
	Experimental	0·52 (0·19–1·44)	0·21	0·38 (0·12–1·19)	0·10	0·60 (0·22–1·63)	0·32	0·41 (0·14–1·27)	0·12
	Regular	2·00 (0·98–4·10)	0·058	1·76 (0·84–3·70)	0·13	1·87 (0·92–3·78)	0·083	1·69 (0·81–3·52)	0·16
Nicotine use									
	Non-use	1 (ref)	..	1 (ref)	..	1 (ref)	..	1 (ref)	..
	Intermittent	2·31 (0·97–5·51)	0·060	2·89 (1·19–7·02)	0·019	2·33 (0·99–5·53)	0·054	2·87 (1·19–6·89)	0·019
	Continued	1·70 (0·93–3·10)	0·082	1·95 (1·03–3·68)	0·039	1·75 (0·96–3·16)	0·066	1·99 (1·07–3·71)	0·030
Alcohol use									
	No use	1 (ref)	..	1 (ref)	..	1 (ref)	..	1 (ref)	..
	Continued	1·28 (0·67–2·43)	0·46	1·70 (0·85–3·41)	0·13	1·28 (0·68–2·41)	0·45	1·63 (0·82–3·22)	0·16
Care intensity at onset									
	Community mental health team	1 (ref)	..	1 (ref)	..	1 (ref)	..	1 (ref)	..
	Crisis team	0·94 (0·26–3·43)	0·93	0·80 (0·21–3·11)	0·75	0·98 (0·27–3·56)	0·97	0·77 (0·20–2·99)	0·71
	Non-compulsory admission	2·40 (1·06–5·39)	0·035	2·24 (0·98–5·14)	0·056	2·59 (1·14–5·87)	0·023	2·43 (1·05–5·59)	0·037
	Compulsory admission	2·17 (0·99–4·72)	0·052	2·08 (0·94–4·62)	0·072	2·44 (1·12–5·34)	0·026	2·32 (1·04–5·16)	0·039
Relationship status									
	In relationship	1 (ref)	..	1 (ref)	..	1 (ref)	..	1 (ref)	..
	Not in relationship	1·49 (0·89–2·50)	0·13	1·78 (1·01–3·14)	0·045	1·53 (0·91–2·58)	0·11	1·89 (1·07–3·34)	0·028
Psychosis diagnosis									
	Non-affective	1 (ref)	..	1 (ref)	..	1 (ref)	..	1 (ref)	..
	Affective	0·73 (0·39–1·37)	0·33	0·90 (0·46–1·74)	0·75	0·78 (0·42–1·46)	0·44	0·98 (0·51–1·88)	0·94
Medication adherence									
	Regular compliance	..	..	1 (ref)	..	..	..	1 (ref)	..
	Irregular compliance	..	..	1·82 (1·05–3·16)	0·032	..	..	1·93 (1·11–3·35)	0·019
	Poor compliance	..	..	4·53 (2·24–9·15)	<0·0001	..	..	4·40 (2·18–8·87)	<0·0001

HRs included adjustment for sociodemographic factors (sex, ethnicity, and relationship status) and clinical factors (psychosis diagnosis, intensity of care at onset, and alcohol, nicotine, and other drug use; with analysis stratified by cannabis use). Where indicated, the model also adjusted for medication adherence. HR=hazard ratio.

* Age of onset was modelled as a restricted cubic spline function with knots placed at ages of onset of 22 years, 25 years, 30 years, and 39 years.

In separate multiple (negative binomial) regression analyses, having a history of stressful life events occurring in the 2 years after the onset of psychosis was significantly associated with the number (incidence rate ratio [IRR] 1·87; 95% CI 1·24–2·80; p=0·0026) and the length (2·53; 1·40–4·67; p=0·0011) of subsequent relapses, compared with not having experienced any stressful life events after psychosis onset (table 2), after controlling for sociodemographic and clinical factors. Including medication adherence in the models did not substantially change the association with the number (1·81; 1·15–2·82; p=0·010) or length (2·30; 1·26–4·32; p=0·0045) of subsequent relapses. Moreover, the number of stressful life events experienced during the 2 years after psychosis onset was significantly associated with the number (1·26; 1·02–1·53; p=0·023) and the length (1·52; 1·12–2·12; p=0·0028) of subsequent relapses (table 3) in models adjusted for sociodemographic and clinical factors. Inclusion of medication adherence in the models did not substantially change the association for the length of subsequent relapses (1·47; 1·10–2·00; p=0·0063), but the association with the number of relapses was no longer significant (1·24; 0·99–1·52; p=0·051). Additional predictors were also found to be significantly associated with relapse and are reported in the appendix (p 9).

**Table 2:** Effect of any stressful life events after onset of psychosis on number and length of subsequent relapses as indexed by hospital admission

		**Number of relapses (n=253)**		**Number of relapses (with medication adherence; n=240)**		**Length of relapses (n=253)**		**Length of relapses (with medication adherence; n=240)**	
		IRR (95% CI)	p value	IRR (95% CI)	p value	IRR (95% CI)	p value	IRR (95% CI)	p value
Any stressful life events before first relapse									
	No	1 (ref)	..	1 (ref)	..	1 (ref)	..	1 (ref)	..
	Yes	1·87 (1·24–2·80)	0·0026	1·81 (1·15–2·82)	0·010	2·53 (1·40–4·67)	0·0011	2·30 (1·26–4·32)	0·0045
Age of onset, years*									
	<22	1 (ref)	..	1 (ref)	..	1 (ref)	..	1 (ref)	..
	22 to <25	0·94 (0·74–1·22)	0·66	0·94 (0·72–1·24)	0·66	0·93 (0·64–1·35)	0·69	1·03 (0·70–1·51)	0·86
	25 to <30	1·18 (0·02–55·0)	0·93	0·76 (0·01–40·7)	0·89	0·87 (0·00–259)	0·96	0·24 (0·00–77·8)	0·60
	30 to <39	0·79 (0·00–19 437)	0·96	3·20 (0·00–123 453)	0·83	1·58 (0·00–4 302 739)	0·95	40·3 (0·00–150 049 840)	0·59
	≥39	1·10 (0·00–3565)	0·98	0·29 (0·00–1273)	0·77	0·78 (0·00–112 259)	0·96	0·06 (0·00–10 706)	0·62
Sex									
	Female	1 (ref)	..	1 (ref)	..	1 (ref)	..	1 (ref)	..
	Male	0·84 (0·57–1·25)	0·39	0·75 (0·50–1·12)	0·15	1·18 (0·67–2·07)	0·53	1·10 (0·62–1·93)	0·72
Ethnicity									
	White	1 (ref)	..	1 (ref)	..	1 (ref)	..	1 (ref)	..
	Asian	1·51 (0·57–3·55)	0·37	0·95 (0·31–2·48)	0·93	1·86 (0·55–6·34)	0·28	1·34 (0·38–4·72)	0·63
	Black African or Caribbean	1·89 (1·17–3·16)	0·012	1·63 (0·99–2·75)	0·063	2·55 (1·37–4·83)	0·0031	2·37 (1·27–4·47)	0·0069
	Mixed	2·05 (0·94–4·23)	0·060	1·78 (0·80–3·75)	0·14	2·28 (0·76–7·20)	0·12	2·03 (0·67–6·39)	0·18
Cannabis use									
	Former	1 (ref)	..	1 (ref)	..	1 (ref)	..	1 (ref)	..
	Never	1·35 (0·72–2·62)	0·36	1·58 (0·82–3·15)	0·18	1·45 (0·63–3·38)	0·38	1·94 (0·81–4·79)	0·13
	Intermittent	1·05 (0·52–2·10)	0·90	1·11 (0·53–2·29)	0·78	2·31 (0·98–5·57)	0·048	2·69 (1·09–6·83)	0·024
	Continued hash-type	1·19 (0·41–3·03)	0·73	1·18 (0·40–3·09)	0·75	0·84 (0·20–3·69)	0·81	0·81 (0·19–3·51)	0·77
	Continued regular skunk-type	1·04 (0·46–2·29)	0·92	1·00 (0·44–2·21)	>0·99	1·51 (0·52–4·52)	0·41	1·45 (0·50–4·34)	0·47
	Continued heavy skunk-type	1·64 (0·86–3·19)	0·14	1·45 (0·73–2·93)	0·29	2·08 (0·82–5·38)	0·10	2·31 (0·89–6·17)	0·063
Other drug use									
	Non-use	1 (ref)	..	1 (ref)	..	1 (ref)	..	1 (ref)	..
	Experimental	0·74 (0·31–1·56)	0·46	0·57 (0·21–1·32)	0·23	1·48 (0·56–4·04)	0·42	1·43 (0·54–3·92)	0·47
	Regular	1·91 (1·06–3·40)	0·029	1·82 (0·99–3·28)	0·050	2·29 (0·95–5·65)	0·051	2·28 (0·96–5·58)	0·052
Nicotine use									
	Non-use	1 (ref)	..	1 (ref)	..	1 (ref)	..	1 (ref)	..
	Intermittent	1·95 (0·85–4·16)	0·095	2·39 (1·04–5·15)	0·032	2·45 (0·93–6·66)	0·066	3·25 (1·23–8·85)	0·017
	Continued	2·00 (1·21–3·31)	0·0069	2·33 (1·37–3·99)	0·0019	2·00 (1·05–3·84)	0·032	2·56 (1·34–4·98)	0·0045
Alcohol use									
	No use	1 (ref)	..	1 (ref)	..	1 (ref)	..	1 (ref)	..
	Continued	1·26 (0·72–2·12)	0·40	1·51 (0·83–2·65)	0·16	1·03 (0·48–2·23)	0·94	1·19 (0·56–2·57)	0·65
Care intensity at onset									
	Community mental health team	1 (ref)	..	1 (ref)	..	1 (ref)	..	1 (ref)	..
	Crisis team	0·99 (0·30–2·98)	0·99	0·87 (0·25–2·72)	0·82	0·92 (0·21–3·83)	0·90	0·82 (0·18–3·58)	0·79
	Non-compulsory admission	2·15 (1·08–4·79)	0·042	1·88 (0·93–4·22)	0·10	2·29 (0·89–6·19)	0·071	2·20 (0·86–5·93)	0·091
	Compulsory admission	2·26 (1·18–4·90)	0·024	2·09 (1·07–4·58)	0·045	4·30 (1·79–10·9)	0·0007	4·76 (1·98–12·2)	0·0003
Relationship status									
	In relationship	1 (ref)	..	1 (ref)	..	1 (ref)	..	1 (ref)	..
	Not in relationship	1·33 (0·87–2·09)	0·20	1·58 (1·00–2·58)	0·057	2·52 (1·36–4·74)	0·0025	3·29 (1·71–6·47)	0·0003
Psychosis diagnosis									
	Non-affective	1 (ref)	..	1 (ref)	..	1 (ref)	..	1 (ref)	..
	Affective	0·74 (0·42–1·23)	0·27	0·91 (0·51–1·56)	0·75	0·48 (0·22–1·03)	0·044	0·55 (0·25–1·21)	0·11
Medication adherence									
	Regular compliance	..	..	1 (ref)	..	..	..	1 (ref)	..
	Irregular compliance	..	..	1·84 (1·17–2·95)	0·010	..	..	1·63 (0·91–2·94)	0·083
	Poor compliance	..	..	3·30 (1·85–5·90)	<0·0001	..	..	2·66 (1·22–5·97)	0·0094

IRRs included adjustment for sociodemographic factors (sex, ethnicity, and relationship status) and clinical factors (psychosis diagnosis, intensity of care at onset, and alcohol, nicotine, and other drug use; with analysis stratified by cannabis use). Where indicated, the model also adjusted for medication adherence. IRR=incidence rate ratio.

* Age of onset was modelled as a restricted cubic spline function with knots placed at ages of onset of 22 years, 25 years, 30 years, and 39 years.

**Table 3:** Effect of number of stressful life events after onset of psychosis on number and length of subsequent relapses as indexed by hospital admission

		**Number of relapses (n=253)**		**Number of relapses (with medication adherence; n=240)**		**Length of relapses (n=253)**		**Length of relapses (with medication adherence; n=240)**	
		IRR (95% CI)	p value	IRR (95% CI)	p value	IRR (95% CI)	p value	IRR (95% CI)	p value
Number of stressful life events before first relapse		1·26 (1·02–1·53)	0·023	1·24 (0·99–1·52)	0·051	1·52 (1·12–2·12)	0·0028	1·47 (1·10–2·00)	0·0063
Age of onset, years*									
	<22	1 (ref)	..	1 (ref)	..	1 (ref)	..	1 (ref)	..
	22 to <25	0·95 (0·74–1·23)	0·68	0·95 (0·73–1·24)	0·71	0·91 (0·63–1·32)	0·62	1·03 (0·70–1·51)	0·88
	25 to <30	1·20 (0·03–55·1)	0·92	0·70 (0·01–36·5)	0·86	1·14 (0·00–352)	0·96	0·27 (0·00–85·0)	0·62
	30 to <39	0·72 (0·00–16 684)	0·95	3·77 (0·00–135 145)	0·80	0·76 (0·00–2 175 338)	0·97	28·6 (0·00–103 185 809)	0·63
	≥39	1·21 (0·00–16,684)	0·96	0·26 (0·00–1090)	0·75	1·44 (0·00–216 033)	0·95	0·09 (0·00–15 318)	0·67
Sex									
	Female	1 (ref)	..	1 (ref)	..	1 (ref)	..	1 (ref)	..
	Male	0·82 (0·56–1·22)	0·33	0·72 (0·48–1·08)	0·11	1·12 (0·64–1·97)	0·66	1·04 (0·59–1·81)	0·89
Ethnicity									
	White	1 (ref)	..	1 (ref)	..	1 (ref)	..	1 (ref)	..
	Asian	1·48 (0·56–3·48)	0·39	0·91 (0·29–2·37)	0·86	1·66 (0·49–5·65)	0·38	1·16 (0·33–4·09)	0·80
	Black African or Caribbean	1·88 (1·16–3·14)	0·012	1·61 (0·98–2·72)	0·068	2·36 (1·26–4·44)	0·0064	2·13 (1·14–4·00)	0·017
	Mixed	1·97 (0·91–4·07)	0·073	1·66 (0·75–3·48)	0·19	1·92 (0·64–5·99)	0·22	1·66 (0·56–5·14)	0·34
Cannabis use									
	Former	1 (ref)	..	1 (ref)	..	1 (ref)	..	1 (ref)	..
	Never	1·39 (0·74–2·69)	0·31	1·59 (0·82–3·14)	0·17	1·54 (0·67–3·59)	0·30	2·12 (0·89–5·20)	0·084
	Intermittent	1·08 (0·53–2·16)	0·83	1·13 (0·54–2·33)	0·75	2·16 (0·90–5·26)	0·072	2·47 (1·00–6·28)	0·040
	Continued hash-type	1·23 (0·42–3·13)	0·68	1·21 (0·41–3·18)	0·71	1·00 (0·24–4·39)	>0·99	0·94 (0·23–4·05)	0·93
	Continued regular skunk-type	1·09 (0·49–2·41)	0·83	1·04 (0·46–2·31)	0·91	1·66 (0·58–4·92)	0·31	1·52 (0·53–4·50)	0·41
	Continued heavy skunk-type	1·72 (0·91–3·33)	0·10	1·48 (0·74–2·99)	0·27	2·14 (0·84–5·54)	0·083	2·34 (0·90–6·22)	0·058
Other drug use									
	Non-use	1 (ref)	..	1 (ref)	..	1 (ref)	..	1 (ref)	..
	Experimental	0·79 (0·33–1·65)	0·55	0·58 (0·21–1·35)	0·25	1·43 (0·54–3·91)	0·47	1·34 (0·51–3·66)	0·55
	Regular	1·83 (1·02–3·24)	0·039	1·74 (0·95–3·13)	0·066	2·05 (0·85–5·07)	0·089	2·05 (0·86–4·98)	0·087
Nicotine use									
	Non-use	1 (ref)	..	1 (ref)	..	1 (ref)	..	1 (ref)	..
	Intermittent	1·98 (0·87–4·22)	0·087	2·39 (1·04–5·13)	0·031	2·66 (1·01–7·28)	0·044	3·69 (1·39–10·1)	0·0077
	Continued	2·00 (1·22–3·30)	0·0067	2·32 (1·37–3·96)	0·0019	2·02 (1·06–3·91)	0·029	2·70 (1·40–5·29)	0·0025
Alcohol use									
	No use	1 (ref)	..	1 (ref)	..	1 (ref)	..	1 (ref)	..
	Continued alcohol	1·24 (0·71–2·08)	0·43	1·45 (0·80–2·53)	0·21	1·07 (0·50–2·32)	0·86	1·23 (0·58–2·65)	0·59
Care intensity at onset									
	Community mental health team	1 (ref)	..	1 (ref)	..	1 (ref)	..	1 (ref)	..
	Crisis team	1·01 (0·30–3·05)	0·98	0·84 (0·24–2·64)	0·78	0·83 (0·19–3·50)	0·80	0·74 (0·16–3·24)	0·69
	Non-compulsory admission	2·27 (1·14–5·06)	0·030	1·97 (0·97–4·42)	0·077	2·33 (0·91–6·29)	0·064	2·23 (0·87–6·01)	0·083
	Compulsory admission	2·42 (1·26–5·23)	0·014	2·20 (1·13–4·83)	0·031	4·24 (1·76–10·8)	0·0007	4·68 (1·94–12·0)	0·0004
Relationship status									
	In relationship	1 (ref)	..	1 (ref)	..	1 (ref)	..	1 (ref)	..
	Not in relationship	1·33 (0·87–2·10)	0·20	1·62 (1·03–2·65)	0·046	2·45 (1·32–4·61)	0·0034	3·34 (1·73–6·58)	0·0002
Psychosis diagnosis									
	Non-affective	1 (ref)	..	1 (ref)	..	1 (ref)	..	1 (ref)	..
	Affective	0·78 (0·44–1·29)	0·36	0·97 (0·54–1·66)	0·93	0·53 (0·25–1·12)	0·077	0·61 (0·28–1·32)	0·18
Medication adherence									
	Regular compliance	..	..	1 (ref)	..	..	..	1 (ref)	..
	Irregular compliance	..	..	1·90 (1·21–3·05)	0·0064	..	..	1·79 (1·01–3·21)	0·037
	Poor compliance	..	..	3·26 (1·83–5·82)	<0·0001	..	..	2·84 (1·31–6·37)	0·0055

IRRs included adjustment for sociodemographic factors (sex, ethnicity, and relationship status) and clinical factors (psychosis diagnosis, intensity of care at onset, and alcohol, nicotine, and other drug use; with analysis stratified by cannabis use). Where indicated, the model also adjusted for medication adherence. IRR=incidence rate ratio.

* Age of onset was modelled as a restricted cubic spline function with knots placed at ages of onset of 22 years, 25 years, 30 years, and 39 years.

Unadjusted fixed-effects analysis (table 4) showed that the risk of relapse was significantly higher during the year in which stressful life events preceded relapse of psychosis (odds ratio 4·33; 95% CI 2·00–9·37; p=0·0002) compared with the year when stressful life events did not precede relapse, and this effect remained significant (3·82; 1·82–8·00; p=0·0004) after controlling for time-varying factors (medication adherence, cannabis use pattern, and other drug use). Furthermore, there was a dose–response relationship between the number of stressful life events and the risk of psychosis relapse such that each unit increase in the number of stressful life events preceding relapse was associated with increased odds of subsequent relapse (1·75; 1·25–2·44; p=0·0010). This effect remained significant (1·62; 1·18–2·21; p=0·0028) in the adjusted analysis. On testing models for the reverse directionality of effect, relapse of psychosis did not increase the risk of exposure to stressful life events or the number of stressful life events in either unadjusted or adjusted models (appendix p 17).

**Table 4:** Fixed-effects logistic regression analysis of stressful life events and risk of relapse

		**Any stressful life events: risk of relapse (unadjusted; n=222)**		**Any stressful life events: risk of relapse (with covariates; n=222)**		**Number of stressful life events: risk of relapse (unadjusted; n=222)**		**Number of stressful life events: risk of relapse (with covariates; n=222)**	
		OR (95% CI)	p value	OR (95% CI)	p value	OR (95% CI)	p value	OR (95% CI)	p value
Any stressful life events before first relapse									
	No	..	..	1 (ref)	..	..	..	..	..
	Yes	4·33 (2·00–9·37)	0·0002	3·82 (1·82–8·00)	0·0004	..	..	..	..
Number of stressful life events before first relapse		..	..	..	..	1·75 (1·25–2·44)	0·0010	1·62 (1·18–2·21)	0·0028
Cannabis use									
	Former or never	..	..	1 (ref)	..	..	..	1 (ref)	..
	Intermittent	..	..	1·71 (0·62–4·71)	0·30	..	..	1·65 (0·60–4·57)	0·33
	Continued	..	..	2·35 (1·25–4·39)	0·0076	..	..	2·23 (1·19–4·16)	0·012
Other drug use									
	Non-use	..	..	1 (ref)	..	..	..	1 (ref)	..
	Experimental	..	..	0·41 (0·11–1·57)	0·19	..	..	0·39 (0·10–1·51)	0·17
	Regular	..	..	1·69 (0·76–3·77)	0·20	..	..	1·73 (0·78–3·87)	0·18
Medication adherence									
	Regular compliance	..	..	1 (ref)	..	..	..	1 (ref)	..
	Irregular compliance	..	..	1·72 (0·93–3·17)	0·082	..	..	1·86 (1·01–3·42)	0·046
	Poor compliance	..	..	4·11 (1·89–8·94)	0·0004	..	..	4·12 (1·89–8·99)	0·0004

These analyses compare the period when an individual was exposed to stressful life events with the period when the same individual was not exposed to stressful life events. ORs adjusted for covariates take into account time-varying factors: medication adherence, cannabis use pattern, and other drug use. OR=odds ratio.

For the number of relapses (appendix p 18), unadjusted fixed-effects analysis showed that significantly more relapses occurred during the year in which stressful life events preceded relapse of psychosis (IRR 2·40; 95% CI 1·41–4·10; p=0·0017) compared with the year when stressful life events did not precede relapse, and this effect remained similar (1·99; 1·24–3·20; p=0·0042) after controlling for time-varying factors (medication adherence, cannabis use pattern, and other drug use). There was also a dose–response relationship between the number of stressful life events and the number of relapses, such that each unit increase in the number of stressful life events experienced preceding relapse was associated with a higher number of relapses (1·41; 1·14–1·75; p=0·0017), and this effect remained significant (1·30; 1·08–1·56; p=0·0053) after adjustment. Again, on testing models for the reverse directionality of effect, the number of relapse events did not increase the risk of exposure to or number of stressful life events in either unadjusted or adjusted models (appendix p 19).

**Figure 2:**
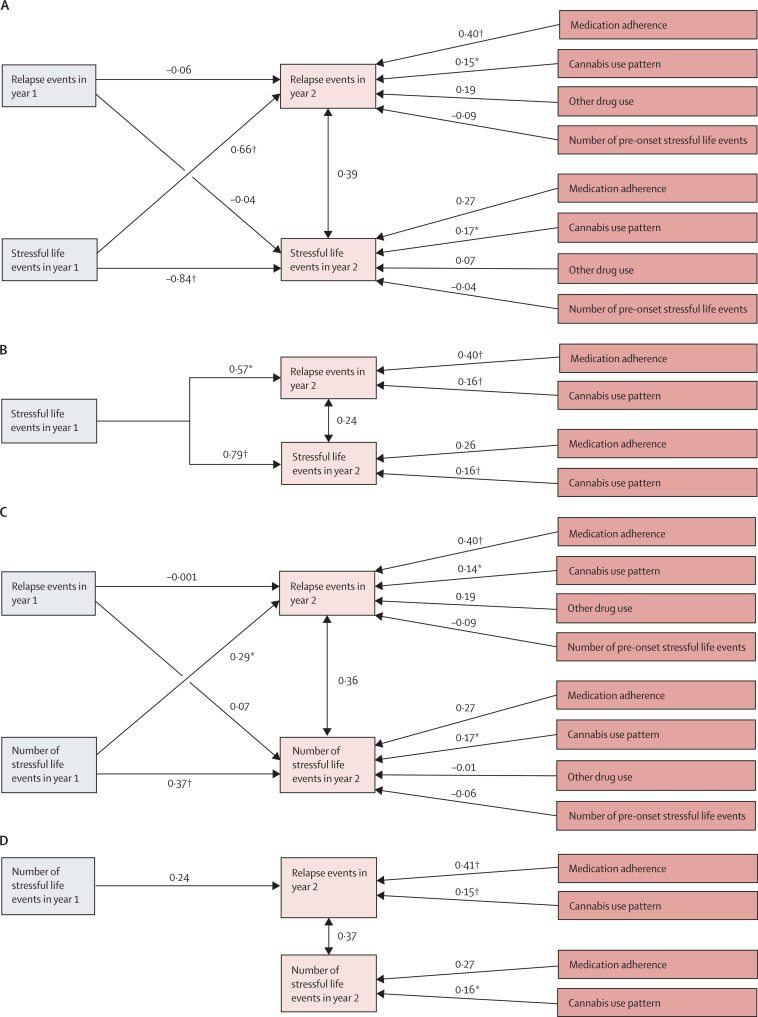
Cross-lagged autoregressive path models examining the directionality of association between relapse and stressful life events Stressful life event status and number of relapse events in the saturated (A) and reduced (B) models, and number of stressful life events and number of relapse events in the saturated (C) and reduced (D) models. Models controlled for medication adherence, cannabis use, other drug use, and pre-onset stressful life events. The saturated path model included all paths to endogenous variables and the reduced model included only statistically significant (p≤0·05) paths. The numbers on the arrows represent regression coefficient estimates (β). *p≤0·05. †p≤0·01.

In the cross-lagged autoregressive path models, exposure to stressful life events (yes or no) during the first year of follow-up had a significant effect (regression coefficient β=0·66, p=0·0055) on the number of relapses occurring during the second year of follow-up, while controlling for the effects of medication adherence, cannabis use, other drug use, and number of stressful life events preceding the psychosis onset (figure 2A, B). The number of relapses within the first year of follow-up did not predict (β=−0·04, p=0·85) subsequent exposure to stressful life events (second year of follow-up), suggesting a unidirectional effect of exposure to stressful life events on psychosis relapse. The number of stressful life events during the first year of follow-up also had a significant effect (β=0·29, p=0·029) on the number of relapses occurring during the second year of follow-up, while controlling for the effects of medication adherence, cannabis use, other drug use, and number of stressful life events preceding the onset of psychosis, indicating a dose–response effect (figure 2C, D). The number of relapses within the first year of follow-up did not predict (β=0·07, p=0·69) the number of stressful life events occurring subsequently (over the second year of follow-up), suggesting a unidirectional effect of exposure to stressful life events on relapse of psychosis.

## Discussion

We used causal inference methods to scrutinise cause–effect relationships between stressful life events and psychosis. Focusing on potential confounders, temporal sequence, and magnitude of association, as well as biological gradient (ie, dose–response effect),^13^ our results provide converging evidence of a causal effect of stressful life events on the risk of relapse in psychosis. We found evidence of a higher adjusted hazard of relapse in individuals exposed to any post-onset stressful life events compared with those unexposed; a greater adjusted hazard of relapse for each additional exposure to post-onset stressful life events (a dose–response effect); a higher incidence and longer duration of psychosis relapse in individuals exposed to any post-onset stressful life events compared with those unexposed; and an increase in the incidence and duration of relapse for each additional exposure to post-onset stressful life events (dose–response). Furthermore, we found a higher risk and greater number of relapses during the year in which stressful life events preceded relapse of psychosis (compared with the year when they did not) and as a function of the number of stressful life events occurring over the same period after controlling for both measured and unmeasured time-invariant confounders, such as shared genetic and familial vulnerability, duration of untreated psychosis, or premorbid adjustment. However, we found no effect of relapse of psychosis or number of relapses on the risk of exposure to or number of stressful life events (ie, no evidence of reverse causation). We also found a significant effect of exposure to and number of stressful life events occurring during the first year of follow-up on the number of psychosis relapses occurring during the subsequent year of follow-up, but no effect of the number of relapses in the first year of follow-up on risk of exposure to or number of stressful life events occurring during the second follow-up year (ie, no evidence of reverse causation).

Collectively, this evidence is consistent with a causal interpretation of the association between stressful life events and relapse of psychosis, and not the reverse, with a similar magnitude but opposite direction of effect to that of antipsychotic medication treatment.^25^ It is also consistent with previous evidence,^10^ experimental research into the effects of stress and the consequences of stress for dopaminergic homoeostasis^26^, ^27^ and psychotic manifestations,^26^ and it is biologically plausible.^28^, ^29^ We also found evidence of an effect of other sociodemographic and clinical risk factors on risk of relapse, consistent with previous literature (appendix p 10).

This study has some limitations, including the retrospective, self-reported assessment of exposure to post-onset stressful life events and confounders, such as medication adherence or pattern of use of various drugs; a lack of consideration of the emotional impact of stressful life events; and that causal inferences are drawn on the basis of observational data. We cannot rule out the possibility of a systematic bias in the recall of stressful life events in those who experienced a psychosis relapse. Future studies could employ real-time monitoring of life events to address these limitations. Because we measured stressful life events based on self-reporting, psychosis-related cognitive impairment might also have affected recall of stressful life events in the present study (see appendix pp 10–11).

A further limitation relates to the use of only the count of stressful life events in our analyses, without accounting for their emotional impact or the individual's capacity to cope. Future studies need to consider individual appraisal of stressful life events, which might meaningfully inform intervention development. Arguably, whether life events are perceived as stressful might depend on an individual's appraisal at a given moment and could be affected by factors such as whether the individual is in receipt of psychotherapeutic intervention (see appendix pp 10–11). Additionally, we used hospital admission as a marker of relapse instead of other markers of outcome, such as symptom severity. Whether a similar association with stressful life events holds true for other indices of relapse, such as worsening of symptoms, needs examination in future studies. We might also have detected evidence of a bidirectional relationship between stressful life events and relapse of psychosis if we had had used a broader definition of life event and relapse of psychosis and carried out more frequent assessments over a longer period of follow-up, as has been found in real-time investigations of the association between stress and psychosis.^30^ Future studies might need to control for potential group differences in clinical remission after the onset of psychosis, because the risk of relapse of psychosis might be higher in those with incomplete remission after the first episode.

The results presented here have important practice and policy implications. They suggest the need to focus on development of interventions at the individual and health-service level that bring together conventional therapies (eg, psychotherapy) and psychoeducation, as well as sensitive awareness campaigns and complementary approaches (eg, lifestyle changes) to help mitigate the harmful effects of stressful life events and prevent psychosis relapse.

## Data sharing

The study investigators own and have complete control of the research data, which can be accessed at any time. For statistical analysis, the data were downloaded and safely stored on a computing system maintained by King's College London. Requests to access de-identified datasets, data dictionaries, and other information from the study should be directed to the corresponding author.

## Declaration of interests

SB has received support from the National Institute for Health Research (NIHR) Clinician Scientist Award (NIHR CS-11-001) and the Medical Research Council (MR/J012149/1). TS has received support from the Wellcome Trust Postdoctoral Fellowship (grant numbers 218641/Z/19/Z). RM has received support from the Biomedical Research Centre, South London and Maudsley NHS Foundation Trust, UK. AEC has received support from the NARSAD Young Investigator Grant awarded by the Brain & Behavior Research Foundation (28336) and funded by the Evelyn Toll Family Foundation. MC has been a consultant or advisor to GW Pharma, GW Pharma Italy, and F Hoffmann-La Roche, outside of this work. All other authors declare no competing interests.
