# Supplementary material for: Stressful life events and relapse of psychosis: analysis of causal association in a 2-year prospective observational cohort of individuals with first-episode psychosis in the UK
Source: Lancet Psychiatry. 2023 Jun;10(6):414–25. doi: 10.1016/S2215-0366(23)00110-4 (PMC10728826; doi:10.1016/S2215-0366(23)00110-4)
Supplement: Supplementary appendix [file mmc1.pdf]

THE LANCET
Psychiatry

Supplementary appendix

This appendix formed part of the original submission and has been peer reviewed. We post it as supplied by the authors.

Supplement to: Bhattacharyya S, Schoeler T, Di Forti M, Murray R, Cullen AE, Colizzi M. Stressful life events and relapse of psychosis: analysis of causal association in a 2-year prospective observational cohort of individuals with first-episode psychosis in the UK. *Lancet Psychiatry* 2023; published online May 2. https://doi.org/S2215-0366(23)00110-4.

Stressful life events and relapse of psychosis: analysis of causal association in a 2-year prospective observational cohort of individuals with first-episode psychosis in the UK

Supplementary Materials

CONTENTS

**Supplementary Methods**	2
**Outcome measures**	2
Psychosis relapse	2
Covariates	2
**Statistical analyses**	5
**Supplementary Results**	9
**Supplementary Discussion 1**	10
**Supplementary Discussion** 2	10
References	12
**Supplementary Figure**	14
**Supplementary Table 1**	15
**Supplementary Table 2**	16
**Supplementary Table 3**	17
**Supplementary Table 4**	18
**Supplementary Table 5**	19

Supplementary Methods

Outcome measures

Psychosis relapse

The various relapse-related outcomes were defined as below.

(i) Risk of relapse: Relapse (yes/no) of psychosis was coded as 'yes' if an individual was admitted to a psychiatric inpatient unit at least once following the onset of illness over the two ensuing years. Any hospital admission that was part of the first episode was not included as a relapse.

(ii) Number of relapses: Number of relapses was calculated by estimating the cumulative number of hospital admissions following the onset of illness over the 2-year period.

(iii) Length of relapse: Length of relapse was calculated by estimating the cumulative number of months spent in hospital over the two years following onset of illness. The time spent in hospital as part of the first episode was not included in this measure.

(iv) Time to first relapse: Time to first relapse was measured as the consecutive number of survival months without experiencing a relapse. Those subjects who did not relapse following the onset were allocated a survival time of 24 months.

Covariates

Demographic and clinical data recorded at onset were used to compare individuals who completed the follow-up assessment from individuals who refused to attend it as well as individuals who presented with a relapse within 2 years from their FEP and individuals who did not. Detailed information about study measures to obtain such information and estimated measurements of all

covariates for such groups has been reported before [1]. Sex (female, male), age of onset, relationship status (in a relationship, not in a relationship), self-reported ethnicity (White, Asian, African, Mixed), other drug use (no use, experimental, regular), alcohol use (no use, user), cannabis use (former user, never user, intermittent user, continued user - hash-type, continued user- regular skunk-type, continued user- heavy skunk-type), cigarette (Nicotine) use (no use, intermittent, continued), care intensity at onset (Community mental health team, Crisis team, Non-compulsory admission, Compulsory admission), medication adherence (Regular compliance, Irregular compliance, Poor compliance), and onset diagnosis (non-affective psychosis, affective psychosis) were assessed and included in the analysis as potential confounders based on previous scientific literature [2]. Diagnosis was assessed based on ICD-10 diagnosis using OPCRIT[3] criteria, classifying subjects into (i) Non-affective psychosis and (ii) Affective psychosis.

Care intensity at onset was included as a proxy measure of illness severity and assessed based on a rating of intensity of service use for each subject at onset: (i) Community mental health team: Required only community treatment without crisis intervention; (ii) Crisis team: Required crisis intervention without hospital admission; (iii) Non-compulsory admission: Required hospital admission without compulsory admission; (iv) Compulsory admission: Required compulsory hospital admission). Compulsory admission referred to admission under various sections of the Mental health Act, UK [section 2, section 3, section 136, through crown and magistrates courts (Section 35, 36, 37, 41 & 48), section 4 or section 5 if converted to sec 2 or 3 subsequently].

Medication adherence was indexed in accordance with previous reports[1], whereby individuals were categorised as (i) Regular compliance: medication not prescribed or good adherence with the prescribed medication (0% - 33% of the time non-adherent) within the two years following the onset of illness; (ii) Irregular compliance: medication prescribed and irregular compliance (34%-66% of the time non-compliant); (iii) Poor compliance: medication prescribed and non-compliance (67%-100% of the time non-compliant).

Cannabis use was assessed using a modified version of the Cannabis Experience Questionnaire (CEQmv)[4], collecting data on premorbid cannabis use, as well as use over the first two years following onset of psychosis. Reliability of the retrospective assessment of cannabis use was assessed by comparing data for n=206 subjects on premorbid cannabis use (ever used before onset) collected at onset of psychosis with data on premorbid cannabis use reported at follow-up. In 92·7% of those compared, reporting of premorbid cannabis use was consistent across both assessments (i.e., at onset and at follow-up). As reported before[1], cannabis use was categorised into the following categories: (i) Former (regular) user: Subjects who had a history of regular cannabis use (defined as use at least once/month for 6 consecutive months) prior to their onset but who used cannabis only infrequently (< 6 times) in the two years following the onset of psychosis; (ii) Never (regular) user: Subjects who were never regular users of cannabis either prior to (less than once/month for 6 consecutive months) or following (< 6 times over the follow-up period) the onset of psychosis; (iii) intermittent user: Subjects who used cannabis more than infrequently (> 6 times) following the onset of psychosis but not consistently every month over the first two years following the onset of illness; (iv) continued user – hash-like: Subjects who used low-potency cannabis ("hash-like" like hash, resin) continuously (defined as use at least once in each month of the years following the onset); (v) continued user – skunk-like (low frequency): Subjects who used high-potency cannabis ("skunk-like") continuously (defined as use at least once in each month of the years following the onset) but in a low-frequency manner (less than daily); and (vi) continued user – skunk-like (high frequency): Subjects who used high-potency cannabis ("skunk-like") continuously in a high-frequency manner (daily use). For the fixed-effects regression and cross-lagged panel analyses, cannabis use pattern was used as a predictor instead of the above by combining the above categories, such that there were 3 levels: (i) Former or Never user (comprising 'Former (regular) user' and 'Never (regular) user'; (ii) Intermittent user (comprising 'intermittent user'); and (iii) Continued user (comprising 'continued user – hash-like', 'continued user – skunk-like (low frequency) and 'continued user – skunk-like (high frequency)'.

Alcohol use (user/ no use) since onset was assessed using CEQmv[4] and the Alcohol Use Disorders Identification Test (AUDIT)[5]. As before[6], subjects were considered as users if they had a history of daily use for at least one month. Other drug use was assessed using CEQmv[4] and defined as use of illicit drugs other than cannabis within two years following the onset of psychosis. It was coded into 3 categories ranging from (i) non-use: No use of illicit drugs other than cannabis in the first two years following the onset of psychosis; (ii) Experimental use: Use (less than 6 times) of illicit drugs other than cannabis in the first two years following onset; (iii) Regular use: Use (6 times or more) of illicit drugs other than cannabis in the first two years following onset. Nicotine use data was collected using the CEQmv[4] and the Nicotine Dependence Scale[7]. Subjects were categorized as (i) non-user: no regular use of nicotine over the first two years following the onset of psychosis; (ii) Intermittent user: intermittent use of nicotine (>2 months of regular use) over the first two years following the onset of psychosis; and (iii) Continued user: continued use of nicotine (>12 months of regular use) over the first two years following the onset of psychosis.

Statistical analyses

Follow-up data for 2 years after the onset of psychosis were modelled for every participant. SLEs were coded as binary categorical variable (yes/no), with those not exposed to SLEs acting as the reference group, as well as count variable (total number of events).

Data analysis was performed using R v4.0.3. Separate survival analyses were carried out using the 'survival' and 'survminer' packages (https://cran.r-project.org/web/packages/survival/index.html; https://cran.r-project.org/web/packages/survminer/index.html) to investigate the effect of any SLEs occurring following the onset of psychosis but before the occurrence of relapse as indexed by hospitalisation and the total number of such SLEs predating the first relapse (occurring within the 2-year period following onset of psychosis) on time to first relapse using Cox proportional hazards regression in a multivariable model controlling for the effect of the aforementioned

sociodemographic and clinical variables [1]. As the proportional hazards assumption was violated at different levels of cannabis use, the model was stratified by cannabis use. Kaplan-Meier plot (created using the 'survminer' package in R) was used to depict unadjusted survival data. Multiple negative binominal regression analyses were employed (using the 'Mass' package; https://cran.r-project.org/web/packages/MASS/index.html) for the count outcome variable (number of relapses) as well as for duration of relapse (which was estimated in months as opposed to days), instead of Poisson regression because of over-dispersion. We have opted for a standard negative binomial regression model instead of a zero-inflated model as the former is more parsimonious and easier to interpret and we did not have any specific rationale to assume that there are two separate processes leading to the zero and non-zero values[8,9]. Further, we checked the performance of the fitted negative binomial models in terms of handling of excess using the 'check_zeroinflation' function in the *performance* package[10] (https://cloud.r-project.org/web/packages/performance/index.html), which did not indicate any substantial underfitting of zeros that invalidated the fitted negative binomial models. Antipsychotic medication adherence was included in separate regression models because this information was available for only a subset of cases, considering that antipsychotic medications were not prescribed for all participants after the onset of psychosis.

To investigate whether the effect of SLEs on subsequent relapse of psychosis was a result of within-person changes over time in exposure to SLEs predating relapse in the first year and the second year after onset of psychosis, as opposed to being an effect of other factors that vary across individuals but do not vary over time and were not measured, such as familial and genetic factors, duration of untreated psychosis, or premorbid adjustment, we employed fixed effect logistic regression analyses for risk of relapse (yes/ no) and fixed effect negative binomial regression analyses for number of relapses (R package lme4 (https://cran.r-project.org/web/packages/lme4/index.html). In those who relapsed, only SLEs that preceded relapse were counted, while in those who did not relapse, all SLEs occurring during the respective time period were counted. We estimated: i) the likelihood of or number of relapses (as indexed by

hospitalisation) during the period when the individual was exposed to the risk factor compared with when the same individual was not exposed to the risk factor; and ii) the effect of number of SLEs by estimating the likelihood of or number of relapses (as indexed by hospitalisation) during the period when the individual was exposed to the risk factor compared with when the same individual was not exposed to the risk factor. For each outcome of interest, we first examined a simple model (unadjusted analysis) and then multiple regression models including pattern of cannabis use (entered as an ordinal variable with 3-levels: (i) Former or Never user (ii) Intermittent user and (iii) Continued user), other drug use (entered as an ordinal variable with 3-levels: (i) non-use, (ii) experimental use and (iii) regular use) and medication adherence (entered as an ordinal variable with 3-levels: (i) regular compliance, (ii) irregular compliance and (iii) poor compliance) as covariates. In fixed-effects analysis, each person becomes their own control, such that only those individuals with a change in exposure over the time period under consideration are selected. Unlike the estimate from a conventional regression analysis, which is a between-person estimate, the estimate from a fixed effects regression is a within-person estimate that takes into account unmeasured personal characteristics that may confound the relationship between the exposure and outcome of interest. In order to investigate the direction of the association between SLEs and relapse of psychosis, we also estimated competing reverse causation models in which we tested the effects of relapse of psychosis and number of relapses on the likelihood and number of SLEs.

To further investigate the direction of the association between SLEs and relapse of psychosis and minimize the influence of reverse causation, we estimated cross-lagged autoregressive path models using the '*sem*' function (R package *lavaan* (https://cran.r-project.org/web/packages/lavaan/index.html) with number of relapses [Relapse in year 1 (R:Y1) and relapses in year 2 (R:Y2)] as the dependent variable and exposure (yes/ no) to SLEs [(SLEs in year 1 (LE:Y1) and SLEs in year 2 (LE:Y2)] or number of SLEs [(number of SLEs in year 1 (No_LE:Y1) and number of SLEs in year 2 (No_LE:Y2)] as the independent variables (in separate models) to examine whether exposure to (or number of) SLEs predicted subsequent relapse and

vice versa (i.e., whether relapse predicted subsequent exposure to SLEs or number of SLEs). All SLEs occurring in the respective periods were counted for these analyses. Models were estimated controlling for medication adherence (entered as an ordinal variable with 3-levels: (i) regular compliance, (ii) irregular compliance and (iii) poor compliance), cannabis use pattern (entered as an ordinal variable with 3-levels: (i) Former or Never user (ii) Intermittent user and (iii) Continued user), other drug use (entered as an ordinal variable with 3-levels: (i) non-use, (ii) experimental use and (iii) regular use) and number of pre-onset SLEs. Models included a lagged path, with R:Y2 and LE:Y2 (or No_LE:Y2) as the dependent variables and LE:Y1 (or No_LE:Y1) and R:Y1 as the corresponding predictors; autoregressive paths estimating the correlation between LE:Y1 (or No_LE:Y1) and LE:Y2 (or No_LE:Y2) and between R:Y2 and LE:Y2; and a part regressing the effect of covariates (medication adherence, cannabis use pattern, other drug use and pre-onset SLEs) on the dependent variables R:Y2 and LE:Y2 (or No_LE:Y2). The cross-lagged path analysis approach that we applied uses general linear models. Variables were entered as ordered variables and weighted least square mean and variance adjusted estimators were used with diagonally weighted least squares (DWLS) to estimate the model parameters and the full weight matrix to compute robust standard errors (because of non-normal data) and a test statistic adjusted for mean and variance. The coefficients represent the standardised regression coefficients (beta). Model goodness of fit was assessed using root mean-squared error of approximation (RMSEA; values $\leq$ 0.05 indicating good fit) and comparative fit index (CFI; values >0.95 indicating good fit) and models with good fit indices are reported. As a first step a saturated path model including all paths to endogenous variables was specified followed by a more parsimonious path model including only statistically significant ($p \leq 0.05$) paths. Regression coefficients have been reported with unidirectional and covariances with bidirectional arrows in the path model figures.

Supplementary Results

There was some evidence that other predictors may be significantly linked to relapse, including cigarette use, other drug use, Black African ethnicity, higher care intensity at onset, not being in a relationship, and medication non-adherence (Tables 1-3). Including medication adherence in such models did not systematically diminish the detrimental effects of the identified additional predictors (Tables 1-3). Finally, male sex and affective psychosis diagnosis at onset were found to be protective towards either hazard (Table 1) or length (Table 2) of relapse.

Supplementary Discussion 1

Several sociodemographic and clinical factors may affect rates of relapses requiring admission among individuals suffering from preexisting psychiatric disorders [11,12]. Among all, higher clinical severity [11,12] and poor medication adherence [13] have been found to be robust indicators of subsequent admissions and poor outcome. Also, evidence supports higher odds of poor outcome among psychosis individuals of non-white ethnic origin [14], not being in a relationship [15], and suffering from a non-affective psychosis [16], with also potential sex-driven differences [17]. Further, a number of studies support a detrimental effect of cigarette use on psychosis outcome, in terms of higher non-remission, possibly by interfering with medication adherence [18]. Psychosis-inducing effects of several illicit drugs are also well-known [19]. In the current study, such factors were all found to play a significant role in causing relapse and prolonging its duration, thus being entirely consistent with previous work.

Supplementary Discussion 2

While we cannot completely rule out the possibility that cognitive impairment may affect the recall of SLEs in people with psychosis, such an effect would have had to be systematically different between those who relapse and those who do not, to have confounded the results presented here. Nevertheless, this underscores the importance of more frequent follow-up assessments to minimize the effect of poor recall while assessing SLEs in people with psychosis. A further limitation relates to use of only the count of SLEs in our analyses without accounting for their emotional impact or the individual's capacity to cope. Future studies need to take into account individual appraisal of SLEs, which may meaningfully inform intervention development. Related to this, it may also be argued that whether life-events are perceived as stressful may depend on their

momentary appraisal and therefore potentially affected by factors such as whether somebody is in receipt of psychotherapeutic intervention. Events captured by the questionnaire have been associated with long-term contextual threat[20]. As such, other factors such as receiving psychotherapeutic intervention may affect them to a lesser extent. Nevertheless, while we cannot be completely certain, such an effect would have had to be systematically different between those who got hospitalized and those who did not for the nature of the SLE construct to have confounded our results. Additionally, we used hospitalization as a marker of relapse instead of other markers of outcome such as symptom severity. Whether a similar relationship with SLEs holds true for other indices of relapse such as worsening of symptoms needs examination in future studies. In a similar vein, it may also be argued that we might have detected evidence of a bidirectional relationship between SLEs and relapse of psychosis had we used a broader definition of life-event and relapse of psychosis and carried out more frequent assessments over a longer period of follow-up, as has been found in real-time investigations of the association between stress and psychosis[21]. Future studies may also need to control for potential group differences in clinical remission following the onset of psychosis, which we did not, as the risk of relapse of psychosis may be higher in those with incomplete remission following the first episode. However, incomplete remission following onset of psychosis would have had to be systematically more likely in those who experience SLEs after the onset of psychosis compared to those who do not, to have confounded the results of the present study. As such, we did not find any evidence from cross-lagged path analyses that relapse of psychosis, an index of poor outcome that may arguably be considered related to incomplete remission, was associated with increased risk of experiencing SLEs subsequently.

References

1. Schoeler T, Petros N, Di Forti M, et al. Effects of continuation, frequency, and type of cannabis use on relapse in the first 2 years after onset of psychosis: an observational study. *Lancet Psychiatry* 2016; **3**(10): 947-53.
2. Martland N, Martland R, Cullen AE, Bhattacharyya S. Are adult stressful life events associated with psychotic relapse? A systematic review of 23 studies. *Psychol Med* 2020; **50**(14): 2302-16.
3. McGuffin P, Farmer A, Harvey I. A polydiagnostic application of operational criteria in studies of psychotic illness. Development and reliability of the OPCRIT system. *Arch Gen Psychiatry* 1991; **48**(8): 764-70.
4. Di Forti M, Marconi A, Carra E, et al. Proportion of patients in south London with first-episode psychosis attributable to use of high potency cannabis: a case-control study. *Lancet Psychiatry* 2015; **2**(3): 233-8.
5. Hodgson RJ, John B, Abbasi T, et al. Fast screening for alcohol misuse. *Addict Behav* 2003; **28**(8): 1453-63.
6. Faber G, Smid HG, Van Gool AR, Wunderink L, van den Bosch RJ, Wiersma D. Continued cannabis use and outcome in first-episode psychosis: data from a randomized, open-label, controlled trial. *J Clin Psychiatry* 2012; **73**(5): 632-8.
7. Fagerstrom KO. Measuring degree of physical dependence to tobacco smoking with reference to individualization of treatment. *Addict Behav* 1978; **3**(3-4): 235-41.
8. Allison P. Do we really need zero-inflated models? 2012. https:// statisticalhorizons.com/zero-inflated-models (accessed 12 March 2023).
9. Schmidt AM, Pereira JBM, Vieira PP. Do we always need a zero inflated model to capture an apparent excess of zeros? April 2008 ed. Rio de Janeiro, RJ, Brazil: Departamento de M´etodos Estat´ısticos, Universidade Federal do Rio de Janeiro; 2008.
10. Lüdecke D, Ben-Shachar M, Patil I, Waggoner P, Makowski D. Performance: An R package for assessment, comparison and testing of statistical models. *J Open Source Softw* 2021; **6**(60): 3139.
11. Colenda CC, Hamer RM. First admission young adult patients to a state hospital: relative risk for rapid readmission. *Psychiatr Q* 1989; **60**(3): 227-36.
12. Wan TT, Ozcan YA. Determinants of psychiatric rehospitalization: a social area analysis. *Community Ment Health J* 1991; **27**(1): 3-16.
13. Alvarez-Jimenez M, Priede A, Hetrick SE, et al. Risk factors for relapse following treatment for first episode psychosis: a systematic review and meta-analysis of longitudinal studies. *Schizophr Res* 2012; **139**(1-3): 116-28.
14. Bhugra D, Leff J, Mallett R, Der G, Corridan B, Rudge S. Incidence and outcome of schizophrenia in whites, African-Caribbeans and Asians in London. *Psychol Med* 1997; **27**(4): 791-8.
15. White R, Haddock G, Campodonico C, Haarmans M, Varese F. The influence of romantic relationships on mental wellbeing for people who experience psychosis: A systematic review. *Clin Psychol Rev* 2021; **86**: 102022.
16. Cerqueira RO, Ziebold C, Cavalcante D, et al. Differences of affective and non-affective psychoses in early intervention services from Latin America. *J Affect Disord* 2022; **316**: 83-90.
17. Giordano GM, Bucci P, Mucci A, Pezzella P, Galderisi S. Gender Differences in Clinical and Psychosocial Features Among Persons With Schizophrenia: A Mini Review. *Front Psychiatry* 2021; **12**: 789179.
18. Colizzi M, Carra E, Fraietta S, et al. Substance use, medication adherence and outcome one year following a first episode of psychosis. *Schizophrenia Research* 2016; **170**(2-3): 311-7.

19. Fiorentini A, Cantù F, Crisanti C, Cereda G, Oldani L, Brambilla P. Substance-Induced Psychoses: An Updated Literature Review. *Front Psychiatry* 2021; **12**: 694863.
20. Brugha T, Bebbington P, Tennant C, Hurry J. The List of Threatening Experiences: a subset of 12 life event categories with considerable long-term contextual threat. *Psychol Med* 1985; **15**(1): 189-94.
21. Klippel A, Schick A, Myin-Germeys I, Rauschenberg C, Vaessen T, Reininghaus U. Modelling the temporal interplay between stress and affective disturbances in pathways to psychosis: an experience sampling study. *Psychol Med* 2022; **52**(13): 2776-85.

Supplementary Figure. Flow diagram showing participants included in analyses

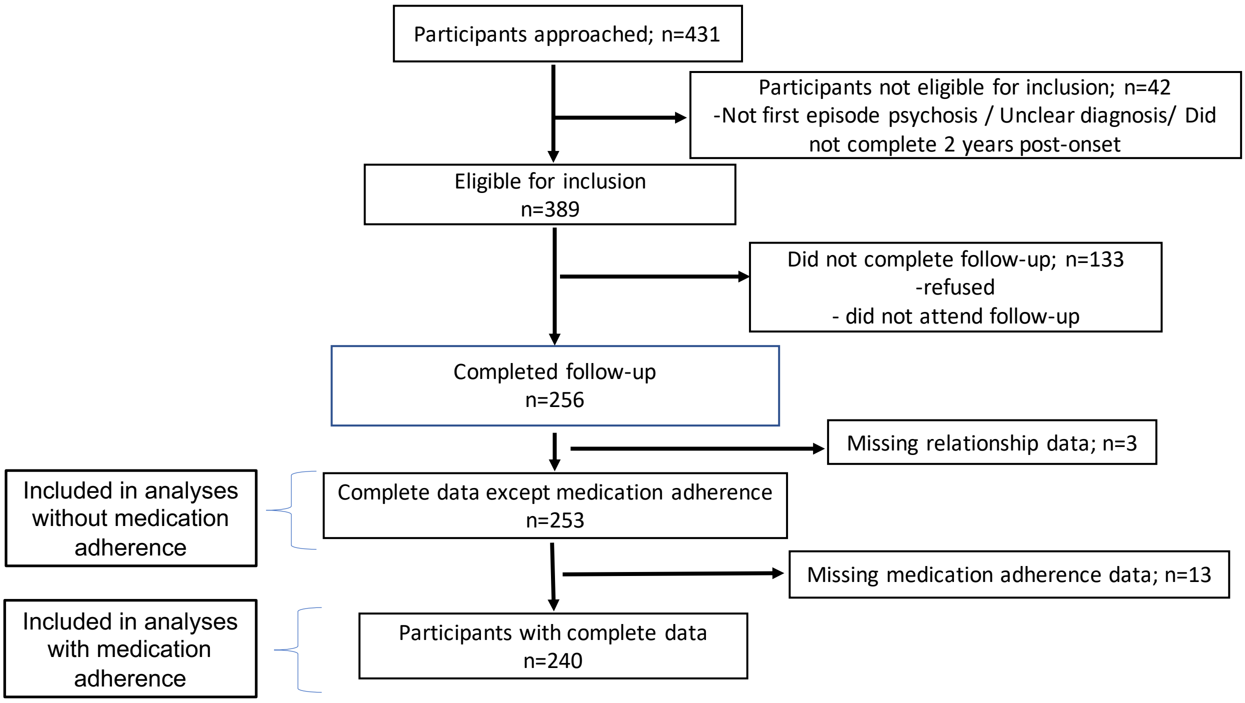

Supplementary Table 1. Patient Characteristics (N = 253)

Variable	Relapse (=No), N=161[1]	Relapse (=Yes), N=92[1]
**Time to first relapse (months)[2]**	24.0 (24.0, 24.0)	11.8 (7.4, 17.5)
**No. of relapses**		
0	161 (100%)	0 (0%)
1	0 (0%)	63 (68%)
2	0 (0%)	22 (24%)
3	0 (0%)	7 (8%)
**Length of relapses (months)**	0.00 (0.00, 0.00)	1.61 (0.98, 2.92)
**Any SLEs before first relapse[2]**	16 (10%)	31 (34%)
**Interval between SLEs and first relapse (months) [2]**	1.8 (0.7, 7.0); Range (0-14.49)	2.1 (1.0, 6.9); Range (0-14.82)
**No. of SLEs before first relapse [2]**		
0	145 (90%)	61 (66%)
1	10 (6%)	19 (21%)
2	1 (1%)	8 (9%)
3	4 (2%)	2 (2%)
4	1 (1%)	2 (2%)
**Cannabis use**		
Former	41 (25%)	12 (13%)
Never	71 (44%)	31 (34%)
Intermittent	21 (13%)	14 (15%)
Continued Hash-type	5 (3%)	4 (4%)
Continued regular Skunk- type	10 (6%)	13 (14%)
Continued heavy Skunk-type	13 (8%)	18 (20%)
**Other drug use**		
non-use other drug	136 (84%)	73 (79%)
experimental other drug	13 (8%)	5 (5%)
regular other drug	12 (7%)	14 (15%)
**Nicotine use**		
non-use nicotine	80 (50%)	29 (32%)
intermittent nicotine	12 (7%)	9 (10%)
continued nicotine	69 (43%)	54 (59%)
**Alcohol Use**		
non-use alcohol	143 (89%)	76 (83%)
User alcohol	18 (11%)	16 (17%)
**Ethnicity**		
White	62 (39%)	22 (24%)
Asian	10 (6%)	6 (7%)
Black African or Carribean	81 (50%)	58 (63%)
Mixed	8 (5%)	6 (7%)
**Care Intensity at onset**		
Community Mental health team	31 (19%)	8 (9%)
Crisis team	13 (8%)	4 (4%)
Non-compulsory admission	49 (30%)	31 (34%)
Compulsory admission	68 (42%)	49 (53%)
**Sex**		
Female	60 (37%)	38 (41%)
Male	101 (63%)	54 (59%)
**Psychosis Diagnosis**		
Non-affective	131 (81%)	78 (85%)
Affective	30 (19%)	14 (15%)
**Age of onset**	26 (22, 32)	25 (21, 32)
**Relationship status**		
in relationship	45 (28%)	21 (23%)
not in relationship	116 (72%)	71 (77%)
**Medication adherence**		
Regular compliance	83 (55%)	23 (26%)
Irregular compliance	54 (36%)	47 (53%)
Poor compliance	14 (9%)	19 (21%)
Missing	10	3

[1]Median (IQR), n (%); [2]For individuals who did not relapse, figures refer to the entire 24-month follow-up period; SLE, Stresssful life events

Supplementary Table 2. Effect of pre-onset stressful life events on hazard of subsequent relapse as indexed by hospitalization

Characteristic	Any SLEs before onset: hazard of relapse (n=253)			No. of SLEs before onset: hazard of relapse (n=253)		
	HR[1]	95% CI[1]	*p*	95% CI[1]	HR[1]	*p*
**Any pre-onset SLEs**	0.94	0.57, 1.55	0.8			
**other drug use**						
1: non-use other drugs	—	—		—	—	
2: experimental other drugs	0.58	0.21, 1.55	0.3	0.57	0.21, 1.52	0.3
3: regular other drugs	1.77	0.87, 3.57	0.11	1.77	0.87, 3.58	0.11
**Nicotine use**						
1: non-use nicotine	—	—		—	—	
2: intermittent nicotine	2.20	0.93, 5.20	0.074	2.07	0.87, 4.95	0.10
3: continued nicotine	1.86	1.03, 3.35	0.039	1.80	0.99, 3.25	0.053
**Alcohol Use**						
1: no-use alcohol	—	—		—	—	
3: user alcohol	1.26	0.67, 2.37	0.5	1.26	0.67, 2.35	0.5
**Ethnicity**						
0: White	—	—		—	—	
1: Asian	1.86	0.68, 5.09	0.2	1.93	0.71, 5.28	0.2
2: Black African or Carribean	2.04	1.15, 3.63	0.015	2.05	1.16, 3.65	0.014
3: Mixed	1.78	0.66, 4.77	0.3	1.79	0.66, 4.82	0.3
**Care Intensity at onset**						
0: Community Mental health team	—	—		—	—	
1: Crisis team	0.92	0.26, 3.28	0.9	0.93	0.26, 3.29	>0.9
2: Non-compulsory admission	2.39	1.07, 5.33	0.033	2.38	1.07, 5.31	0.034
3: Compulsory admission	2.27	1.05, 4.90	0.036	2.25	1.05, 4.84	0.038
**Sex**						
0: Female	—	—		—	—	
1: Male	0.72	0.45, 1.15	0.2	0.70	0.44, 1.12	0.14
**Psychosis Diagnosis**						
0: Non-affective	—	—		—	—	
1: Affective	0.77	0.41, 1.46	0.4	0.78	0.42, 1.46	0.4
**Age of onset**[2]						
<22 years	—	—		—	—	
22 - <25 years	1.01	0.73, 1.40	>0.9	1.02	0.74, 1.41	>0.9
25 - <30 years	0.41	0.00, 44.5	0.7	0.39	0.00, 43.6	0.7
30 - <39 years	16.0	0.00, 2,898,116	0.7	17.4	0.00, 3,287,156	0.6
≥39 years	0.08	0.00, 1,135	0.6	0.07	0.00, 1,086	0.6
Relationship status						
1: in relationship	—	—		—	—	
2: not in relationship	1.45	0.87, 2.43	0.2	1.42	0.84, 2.39	0.2
**Number of pre-onset SLEs**				0.93	0.82, 1.07	0.3

[1] HR = Hazard Ratio, CI = Confidence Interval; SLEs, Stressful life events
[2] Age of onset was modelled as a restricted cubic spline function with knots placed at ages of onset of 22, 25, 30 and 39 years.

Supplementary Table 3. Effects of risk of relapse on stressful life events: Fixed-effects regression models

Characteristic	Risk of relapse: Effect on SLEs risk (n=222)			Risk of relapse: Effect on SLEs risk (with covariates) (n=222)			Risk of relapse: Effect on no. of SLEs (n=222)			Risk of relapse: Effect on no. of SLEs (with covariates) (n=222)		
	OR[1]	95% CI[1]	p-value	OR[1]	95% CI[1]	p-value	IRR[1]	95% CI[1]	p-value	IRR[1]	95% CI[1]	p-value
**Risk of relapse**	2.38	0.05, 105	0.7	1.45	[2]Not estimable	>0.9	1.08	0.67, 1.75	0.8	1.10	0.65, 1.86	0.7
**Cannabis use pattern**												
Former/ Never user				—	—					—	—	
Intermittent user				7.83	[2]Not estimable	0.9				0.96	0.01, 103	>0.9
Continued user				3.85	[2]Not estimable	>0.9				0.82	0.01, 83.7	>0.9
**Medication adherence**												
Regular compliance				—	—					—	—	
Irregular compliance				0.96	[2]Not estimable	>0.9				3.99	0.05, 294	0.5
Poor compliance				0.00	[2]Not estimable	0.086				0.97	0.00, 2,411	>0.9
**Other drug use**												
non-use other drugs				—	—					—	—	
experimental other drugs				0.00	[2]Not estimable	>0.9				0.00	0.00, Inf	>0.9
regular other drugs				0.00	[2]Not estimable	0.6				2.16	0.01, 719	0.8

[1] OR = Odds Ratio, IRR = Incidence Rate Ratio, CI = Confidence Interval
[2] Variance-covariance matrices not positive definite or contained NA values; SLEs, Stressful life events

Supplementary Table 4. Effects of stressful life events on number of relapses: Fixed-effects negative binomial regression

**Characteristic**	**Any SLEs: No. of relapses (n=222)**			**Any SLEs: No. of relapses (with covariates) (n=222)**			**No. of SLEs: No. of relapses (n=222)**			**No. of SLEs: No.of relapses (with covariates) (n=222)**		
	**IRR[1]**	**95% CI[1]**	**p-value**	**IRR[1]**	**95% CI[1]**	**p-value**	**IRR[1]**	**95% CI[1]**	**p-value**	**IRR[1]**	**95% CI[1]**	**p-value**
**Any SLE before first relapse**												
No	—	—		—	—							
Yes	2.40	1.41, 4.10	0.0017	1.99	1.24, 3.20	0.0042						
**No. of SLEs before first relapse**							1.41	1.14, 1.75	0.0016	1.30	1.08, 1.56	0.0053
**Cannabis use pattern**												
Former/ Never user				—	—					—	—	
Intermittent user				1.34	0.63, 2.88	0.4				1.34	0.62, 2.89	0.5
Continued user				1.50	0.95, 2.37	0.085				1.47	0.93, 2.33	0.10
**Medication adherence**												
Regular compliance				—	—					—	—	
Irregular compliance				1.90	1.18, 3.07	0.009				1.96	1.22, 3.15	0.005
Poor compliance				2.88	1.65, 5.03	<0.001				2.91	1.66, 5.09	<0.001
**Other drug use**												
non-use other drugs				—	—					—	—	
experimental other drugs				0.45	0.14, 1.46	0.2				0.44	0.13, 1.43	0.2
regular other drugs				1.60	0.94, 2.74	0.084				1.59	0.93, 2.74	0.092

[1] IRR = Incidence Rate Ratio, CI = Confidence Interval; SLEs, Stressful life events

Supplementary Table 5. Effects of number of relapses on stressful life events: Fixed-effects regression models

Characteristic	No. of relapses: Effect on SLEs risk (n=222)			No. of relapses: Effect on SLEs risk (with covariates) (n=222)			No. of relapses: Effect on No. of SLEs (n=222)			No. of relapses: Effect on No. of SLEs (with covariates) (n=222)		
	OR[1]	95% CI[1]	p-value	OR[1]	95% CI[1]	p-value	IRR[1]	95% CI[1]	p-value	IRR[1]	95% CI[1]	p-value
**No. of relapses**	1.72	0.11, 27.2	0.7	1.49	[2]Not estimable	>0.9	1.05	0.74, 1.49	0.8	1.05	0.72, 1.54	0.8
**Cannabis use pattern**												
Former/ Never user				—	—					—	—	
Intermittent user				1.30	[2]Not estimable	>0.9				0.93	0.01, 102	>0.9
Continued user				0.73	[2]Not estimable	>0.9				0.83	0.01, 86.7	>0.9
**Medication adherence**												
Regular compliance				—	—					—	—	
Irregular compliance				3.78	[2]Not estimable	>0.9				3.99	0.05, 300	0.5
Poor compliance				0.83	[2]Not estimable	>0.9				0.97	0.00, 2,526	>0.9
**Other drug use**												
non-use other drugs				—	—					—	—	
experimental other drugs				0.00	[2]Not estimable	>0.9				0.00	0.00, Inf	>0.9
regular other drugs				1.75	[2]Not estimable	>0.9				2.15	0.01, 739	0.8

[1] OR = Odds Ratio, IRR = Incidence Rate Ratio, CI = Confidence Interval
[2] Variance-covariance matrices not positive definite or contained NA values; SLEs, Stressful life events
